# Variable Expression of GLIPR1 Correlates with Invasive Potential in Melanoma Cells

**DOI:** 10.3389/fonc.2013.00225

**Published:** 2013-08-30

**Authors:** Anshul Awasthi, Adele G. Woolley, Fabienne J. Lecomte, Noelyn Hung, Bruce C. Baguley, Sigurd M. Wilbanks, Aaron R. Jeffs, Joel D. A. Tyndall

**Affiliations:** ^1^School of Pharmacy, University of Otago, Dunedin, New Zealand; ^2^Department of Pathology, Dunedin School of Medicine, University of Otago, Dunedin, New Zealand; ^3^Department of Biochemistry, Otago School of Medical Sciences, University of Otago, Dunedin, New Zealand; ^4^Auckland Cancer Society Research Centre, The University of Auckland, Auckland, New Zealand

**Keywords:** GLIPR1, melanoma, invasion, methylation, CAP

## Abstract

GLI pathogenesis-related 1 (GLIPR1) was previously identified as an epigenetically regulated tumor suppressor in prostate cancer and, conversely, an oncoprotein in glioma. More recently, GLIPR1 was shown to be differentially expressed in other cancers including ovarian, acute myeloid leukemia, and Wilms’ tumor. Here we investigated GLIPR1 expression in metastatic melanoma cell lines and tissue. GLIPR1 was variably expressed in metastatic melanoma cells, and transcript levels correlated with degree of GLIPR1 promoter methylation *in vitro*. Elevated GLIPR1 levels were correlated with increased invasive potential, and siRNA-mediated knockdown of GLIPR1 expression resulted in reduced cell migration and proliferation *in vitro*. Immunohistochemical studies of melanoma tissue microarrays showed moderate to high staining for GLIPR1 in 50% of specimens analyzed. GLIPR1 staining was observed in normal skin in merocrine sweat glands, sebaceous glands, and hair follicles within the dermis.

## Introduction

GLI pathogenesis-related 1 (*GLIPR1*) has been reported to act as a tumor suppressor gene that is down-regulated in prostate cancer (1–3). In contrast, GLIPR1 is up-regulated in glioma (4, 5) and Wilms’ tumor (6) compared to normal tissue, and has recently been shown to be differentially expressed in ovarian cancer cell lines (7). *GLIPR1* encodes a 266 amino acid (∼30 kDa) member of the CAP superfamily (cysteine rich secretory proteins, antigen 5, and pathogenesis-related 1 proteins) (8). The N-terminus of most of the CAP proteins includes a putative signal peptide indicating this protein is secreted or surface exposed. GLIPR1 is characterized by a postulated signal peptide and a putative C-terminal transmembrane domain (TMD) (5).

*GLIPR1* has been reported to be regulated by p53 in prostate cancer (1). Increased apoptosis, accompanied by decreased tumor progression and metastasis were reported following adeno-viral delivery of *Glipr1* in an orthotopic model for metastatic prostate cancer (9). *Glipr1*-mediated proapoptotic activity was also shown to be related to the presence of the N-terminal signal peptide. Li et al. showed that GLIPR1 up-regulation resulted in elevated reactive oxygen species production, leading to apoptosis through activation of the c-Jun–NH_2_ kinase signaling cascade (10). GLIPR1 has also been shown to regulate growth, survival, and invasion of glioma cells (11). Epigenetics studies have shown that hypermethylation in the promoter region of *GLIPR1* is responsible for the down-regulation of GLIPR1 in prostate cancer (2). In addition, methylation studies of *GLIPR1* showed significant hypomethylation in Wilms’ tumor relative to normal tissue (6). In the development of malignant melanoma, epigenetic changes are emerging as important factors where more than 70 hypermethylated genes have been identified and hypomethylation occurs globally in tumor cells [reviewed in (12)].

Despite a growing body of literature pointing to a role for GLIPR1 in cancer, little is known of the normal function of GLIPR1 and of how disruption might contribute to cancer initiation or progression. We identified *GLIPR1* as part of a gene expression signature that predicted invasive potential in melanoma cell lines (13). Here we report on the role of GLIPR1 in melanoma in more detail, and confirm that GLIPR1 is variably expressed in melanoma cells, which is underpinned by differential promoter methylation, and that GLIPR1 levels correlated with invasive potential. We also show that GLIPR1 is variably expressed in melanoma tissue samples, and can be detected in certain adnexal structures of normal epidermis. We also show that GLIPR1 is a glycosylated transmembrane protein transported to the cell surface.

## Materials and Methods

### Cell lines

Melanoma cell lines used for this study were generated from pathologically confirmed metastatic melanoma samples obtained with ethical approval as previously described (14, 15) and cultured in MEM-α (Invitrogen) supplemented with 0.1% insulin-transferrin-sodium selenite (Roche) and 10% fetal bovine serum (FBS; Bio International, New Zealand). Glioma cell lines U251 and SNB75 were obtained from the Developmental Therapeutics Program, National Cancer Institute and cultured in DMEM (Invitrogen) supplemented with 10% FBS. All new cell lines were maintained at 37°C in a humidified atmosphere of 5% CO_2_. The NZM cell lines used for migration assays in this study were chosen based on their classification as either having a lower (NZM12, 15, 45) or higher (NZM9, 40) invasive potential based on previously published transcript and phenotype profiling (13). Given the established association in the literature between elevated GLIPR1 levels and glioma progression, glioma cell lines were included as comparative high GLIPR1 positive controls in this study.

### RNA isolation and real-time reverse transcription quantitative PCR

Total RNA was extracted from cultured cells using RNeasy columns (Qiagen) according to the manufacturer’s specification. Total RNA (100 ng) was transcribed into cDNA using SuperScript III Reverse Transcriptase (Invitrogen), primed with random hexamers (Invitrogen) and oligo d(T) (Invitrogen) in a 20 μL reaction volume according to the manufacturer’s instructions. Transcript abundance was measured using Platinum SYBR Green qPCR SuperMix-UDG with ROX reference dye (Invitrogen) on an ABI 7300 Real-Time PCR System. Reverse transcription quantitative PCR (RT-qPCR) reactions were performed in duplicate with 2.5 ng template cDNA (RNA equivalent) per 20 μL reaction and corresponding no-template controls. Cycling conditions were 50°C for 2 min, 95°C for 2 min, then 40 cycles of 95°C for 15 s/60°C for 1 min, followed by melting curve analysis. *GLIPR1* abundance was normalized to Tyrosine 3-monooxygenase/tryptophan 5-monooxygenase activation protein, zeta polypeptide (YWHAZ), and Ubiquitin C (UBC) reference gene expression and expressed relative to *GLIPR1* levels in NZM15 (possessing the lowest level of *GLIPR1*) using qBase software and the delta–delta-*C*_q_ method.

The following primers were used in RT-qPCR experiments. GLIPR1 forward primer: AGT TCC GAT CAG AGG TGA AAC C; GLIPR1 reverse primer: GCT TCA GCC GTG TAT TAT GTG A; UBC forward primer: ATT TGG GTC GCG GTT CTT G; UBC reverse primer: TGC CTT GAC ATT CTC GAT GGT (RTPrimerDB ID 8); YWHAZ forward primer: ACT TTT GGT ACA TTG TGG CTT CAA; YWHAZ reverse primer: CCG CCA GGA CAA ACC AGT AT (RTPrimerDB ID 9) (16).

### Cell lysate preparation and western blotting

Lysates were prepared by incubating cells for 30 min in buffer containing 50 mM Tris (pH 8.0), 150 mM NaCl, 0.5% (w/v) sodium deoxycholate, 1% (w/v) nonyl phenoxylpolyethoxylethanol (NP40), 0.1% (w/v) sodium dodecyl sulfate (SDS), 20× complete protease inhibitor (50 μl; Roche), 1 μM sodium orthovanadate, and 1 mM phenylmethylsulfonyl fluoride (PMSF). Protein concentration of the lysate was determined using a bicinchoninic acid assay (BCA kit; Pierce Chemical Co.) according to the manufacturer’s instructions. Total protein was resolved by SDS-PAGE and electro transferred onto Bio Trace Polyvinylidene Fluoride (PVDF) transfer membrane (Pall Corporation, pore size 0.45 μm). The PVDF membrane was blocked with 5% (w/v) non-fat milk powder in Tris-Buffered Saline Tween-20 (TBST) followed by staining with mouse anti-GLIPR1 polyclonal antibody (Abnova, H00011010-A01) overnight at 4°C. The specific bands were detected using goat anti-mouse IgG horseradish peroxidase conjugated secondary antibody (Sigma-Aldrich). Immuno-reactive bands were visualized using Super Signal West Pico chemiluminescent substrate (Thermo Fisher Scientific) and exposing the blots to imaging film (Kodak MXB X-Ray film).

The quantification of non-saturated, developed western blots was carried out using a GS-700 Imaging Densitometer (BioRad) and the intensities of individual bands were quantified using BioRad Quantity One software.

### Small interfering RNA transfection

siRNA-mediated knockdown of GLIPR1 was performed using reverse transfection with Lipofectamine RNAiMAX (Invitrogen) and pre-designed siRNAs targeting *GLIPR1* (ON-TARGET*plus* SMARTpool, L-019819-00-0020, Dharmacon) according to the manufacturer’s instructions, with a final siRNA concentration of 10 nM. GLIPR1 knockdown was confirmed using RT-qPCR (24 h) and western blotting (72 h) post-transfection. Negative control experiments were performed using an ON-TARGET*plus* Non-Targeting Pool (D-001810-10-20, Dharmacon). Sense-strand *GLIPR1* siRNA target sequences were:

GAG ACC AAG UGA AAC GUU A; GCU CAA GUA CCC UAA UUU A; UAG CCU GGA UGG UUU CUU U; UGG CUG CGC AGU UCA AUU U

ON-TARGET*plus* GAPD control siRNA was used as a positive control to assess transfection efficiency (D-001830-01, Dharmacon).

### Cell proliferation

Cell proliferation and viability was quantified using an MTT-based cell proliferation kit (Roche) according to the manufacturer’s instructions. Briefly, 2.5 × 10^3^ cells per well of a 96-well plate, in a final volume of 100 μl media were transfected with siRNA (see above). The cells were grown at 37°C, 5% CO_2_, for 2, 4, 6, or 8 days after which they were treated with 10 μl MTT and incubated at 37°C for 4 h. The resulting formazan crystals were then solubilized with 100 μl solubilization solution and incubated overnight at 37°C. The absorbance (570 nm) was then measured with a microplate reader (Molecular Devices). Media was changed every 3 days.

### Transwell migration and invasion assay

Boyden chamber migration assays were carried out in 24-well format using Transwell cell culture inserts with a polyethylene terephthalate (PET) membrane filter and an 8 μm pore size (BD Biosciences). Cell suspension in 200 μL media supplemented with 2% FBS (v/v) was added to the upper chamber (5 × 10^5^ cells/ml). The lower chamber was filled with 600 μl medium supplemented with 10% (v/v) FBS as a chemoattractant. The cells were allowed to migrate across the membrane for 24 h at 37°C, 5% CO_2_. After 24 h, cells were rinsed with PBS and fixed with pre-chilled methanol for 10 min followed by rinsing with distilled H_2_O. Fixed cells were stained with hematoxylin for 5–10 min at room temperature then rinsed with dH_2_O. Cells that remained on the top side of the membrane were removed with a cotton swab, and the remaining cells imaged using an Olympus IX71 inverted microscope. Twenty-five random fields of view were captured per Transwell insert, and the number of cells that had migrated to the bottom side of the membrane was counted by using ImageJ software (17).

Invasion assays were carried out in a similar way using BioCoat Matrigel invasion chambers (BD Biosciences) consisting of a BD Falcon Cell Culture Insert with an 8 μm pore PET membrane, uniformly coated with BD Matrigel Matrix.

To investigate the effect of siRNA-mediated knockdown of GLIPR1 on the migration or invasion of cells, 3 × 10^4^ cells/well were transfected with siRNA targeting *GLIPR1*, or non-targeting siRNA, for 24 h. Cells were trypsinized, pooled, and counted from three to four wells 24 h post-transfection, and 1 × 10^5^ cells were seeded per insert for migration or invasion assays as described above.

### Methylation

*GLIPR1* promoter methylation status was analyzed by using bisulfite genomic sequencing. Genomic DNA was isolated from cultured cells using a Purelink Genomic DNA kit (Invitrogen) according to the manufacturer’s instructions. Genomic DNA (300 ng) was bisulfite-converted and purified by using EZ DNA Methylation-Gold (Zymo Research) according to the manufacturer’s instructions. One microliter of bisulfite-converted DNA was amplified by using two rounds of PCR, purified with a Purelink PCR Purification kit (Invitrogen), then visualized and quantitated by using an Agilent 2100 Bioanalyzer. Purified PCR products were then submitted to the Genetic Analysis Services, University of Otago, for Sanger sequencing. DNA methylation analysis was performed using BiQ Analyzer v2.00 (18). We amplified a 320 bp region using primers for amplification and sequencing of GLIPR1 from bisulfite-treated DNA previously published by Muller et al. (19). This region extends from −104 to −424 of the GLIPR1 translation start site, and corresponds to the CpG’s referred to as “A–D” in (2). The primers were: forward, TTA TTA TGT GTT GAT ATG ATT TTA AAA AG; reverse AAC CCA CAA CTT TAC AAA CC TAA CC.

### Tissue samples and arrays

Skin specimens used in this study were archival human tissue specimens maintained by Healthlab Otago, Dunedin Public Hospital, Dunedin, New Zealand. All specimens were ethically consented for use under approval MEC/07/05/065 (with written informed consent from Multi-Region Ethics Committee, Ministry of Health, New Zealand). Melanoma tissue microarrays were purchased from US Biomax, Inc. Melanoma and nevi tissue microarrays (catalog number ME1001) consisted of 56 cases of malignant melanoma, 20 cases of metastatic melanoma, and 24 cases of benign nevus.

### Immunohistochemistry

Five micrometer paraffin-embedded tissue sections were de-waxed in xylene and rehydrated through a series of graded ethanols. Endogenous peroxidase activity of the tissue was then quenched with 3% (v/v) H_2_O_2_ in methanol for 10 min. Slides were washed with distilled water prior to microwave-mediated antigen retrieval in 10 mM sodium citrate with 0.05% Tween-20, pH 6.0 for 15 min. Cooled sections were rinsed in PBS, blocked with 10% normal goat serum in PBS for 30 min and incubated with a mouse polyclonal anti-GLIPR1 antibody (Abnova, A01; 1:200) overnight at 4°C. The signal was subsequently detected using Vectastain Elite ABC peroxidase-based detection system (Vector Laboratories). For melanoma and skin specimens NovaRED™ (Vector Laboratories) was used for visualization of immuno-reactivity to distinguish from melanin. To verify the specificity of the immuno-reactions, some sections were incubated in normal goat serum instead of primary antibody. Negative control incubations using the same secondary antibody, but omitting the primary antibody were also carried out and showed negative staining. Validation of the antibodies is provided in supplementary material (Figure S1 in Supplementary Material).

All tissue sections were observed and photographed using a Zeiss MC100 camera coupled to a Zeiss Axioplan universal microscope at a power of 200× or 400×. Randomly selected two to three microscopic measuring fields were analyzed for staining and identification of specific cell types. At least 100 cells were counted per microscopic field visualized.

### Statistical analysis

The results are presented as the average values ± standard error of mean (SEM). Data were analyzed using ANOVA and a Student’s *t* test (with unequal variances). All graphs were generated by using Prism 4 (GraphPad Software, Inc.).

## Results

### GLIPR1 expression correlated with promoter methylation and invasive potential *in vitro*

**Figure 1:**
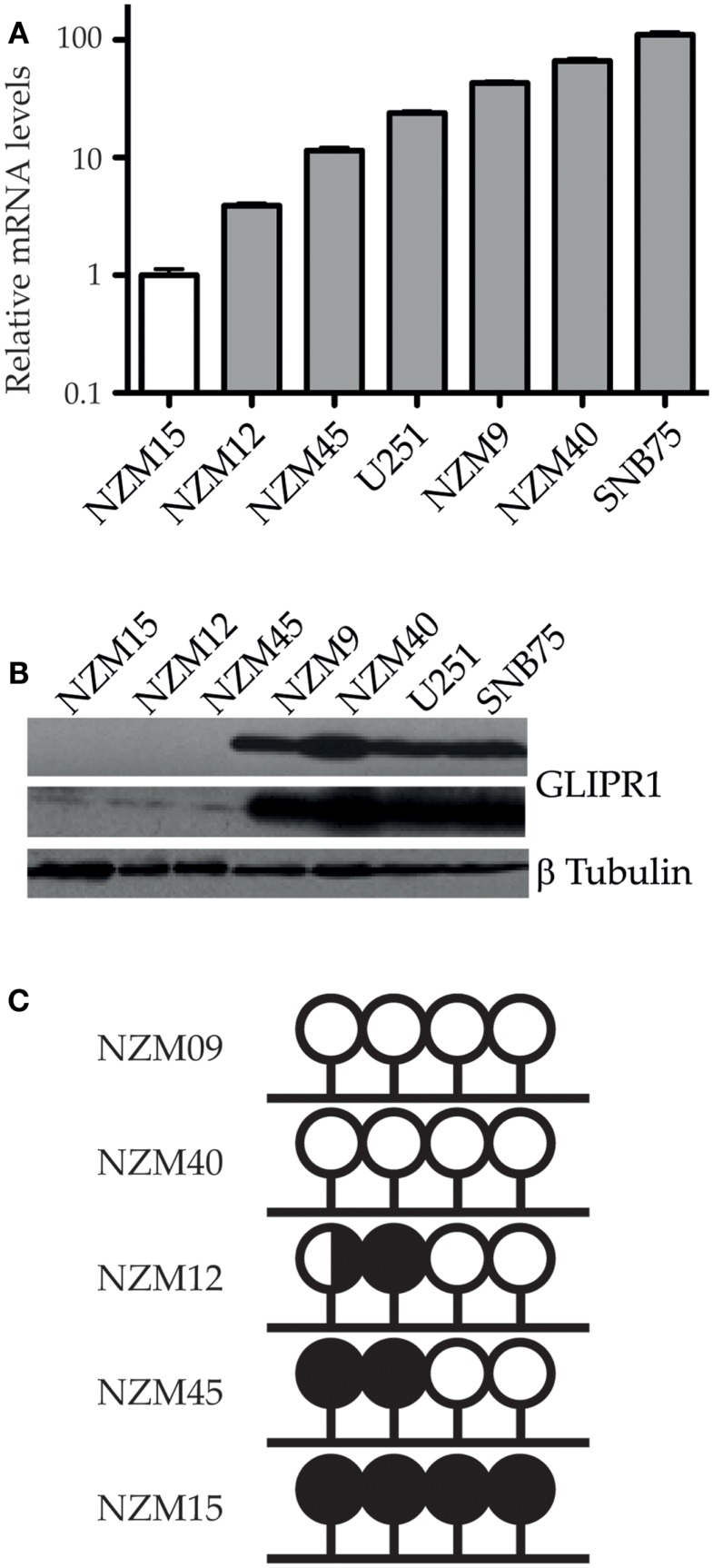
**GLIPR1 expression and promoter methylation in melanoma cells**. **(A)** Relative mRNA transcript levels of GLIPR1 in different melanoma and glioma cells were quantified by RT-qPCR, normalized to reference genes YWHAZ and UBC, and reported relative to levels in NZM15. Results are mean of three independent experiments and error bars indicate SEM. **(B)** GLIPR1 protein levels in different melanoma and glioma cell lines were determined by western blotting with each lane loaded with 100 μg of total protein; the top GLIPR1 blot and β-tubulin loading control (bottom) was exposed for 5 min, and the center GLIPR1 blot exposed overnight. Two separate experiments gave similar results. **(C)** Summary of bisulfite sequencing data showing GLIPR1 CpG promoter methylation status for melanoma cell lines with known invasive potential (this study). NZM09 and NZM40 are strongly invasive compared to the remaining cell lines. Lollipops represent individual CpG dinucleotides within a CpG island in the GLIPR1 promoter. DNA from three different vials of each NZM cell line was sequenced at least twice on both strands. White, unmethylated; black, methylated; black/white, hemimethylated.

*GLIPR1* levels measured using RT-qPCR corresponded with the relative levels reported by previous microarray profiling of NZM cells lines (13), with NZM9 and 40 having relatively higher GLIPR1 abundance compared to NZM12, 15, and 45. Among the five melanoma cell lines, NZM15 cells had the lowest level of *GLIPR1* expression and was used as the baseline reference. The NZM40 cells had the highest *GLIPR1* expression (∼66-fold higher expression relative to NZM15, Figure 1A). NZM9 cells showed a greater *GLIPR1* expression level than that seen in U251 cells which were used as a positive control (∼43 and 24-fold higher than NZM15 respectively). SNB75 cells previously shown to have elevated *GLIPR1* expression (5), showed the highest expression levels within the melanoma and glioma cells tested, with 110-fold higher abundance relative to NZM15 cells. Consistent with the RT-qPCR results, NZM9, NZM40, U251, and SNB75 cells showed higher amounts of GLIPR1 protein, whereas GLIPR1 was undetectable in NZM12, NZM15, and NZM45 after exposure for 5 min and only a very small amount was evident following exposure overnight (Figure 1B). Variable GLIPR1 transcript levels were associated with differences in promoter methylation in a panel of melanoma cell lines of known invasive potential (Figure 1C; Figure S2A in Supplementary Material), with increasing promoter methylation associated with decreasing GLIPR1 abundance (*r*^2^ = 0.82, *p* = 0.037). When additional cell lines from a previous study (13) were included in the analysis the correlation improved (*r*^2^ = 0.83, *p* = 0.002; Figures S2B,C in Supplementary Material). Demethylation treatment with 5-azacytidine caused increased transcript abundance in NZM15, NZM12, and NZM45 (Figure S3 in Supplementary Material).

**Figure 2:**
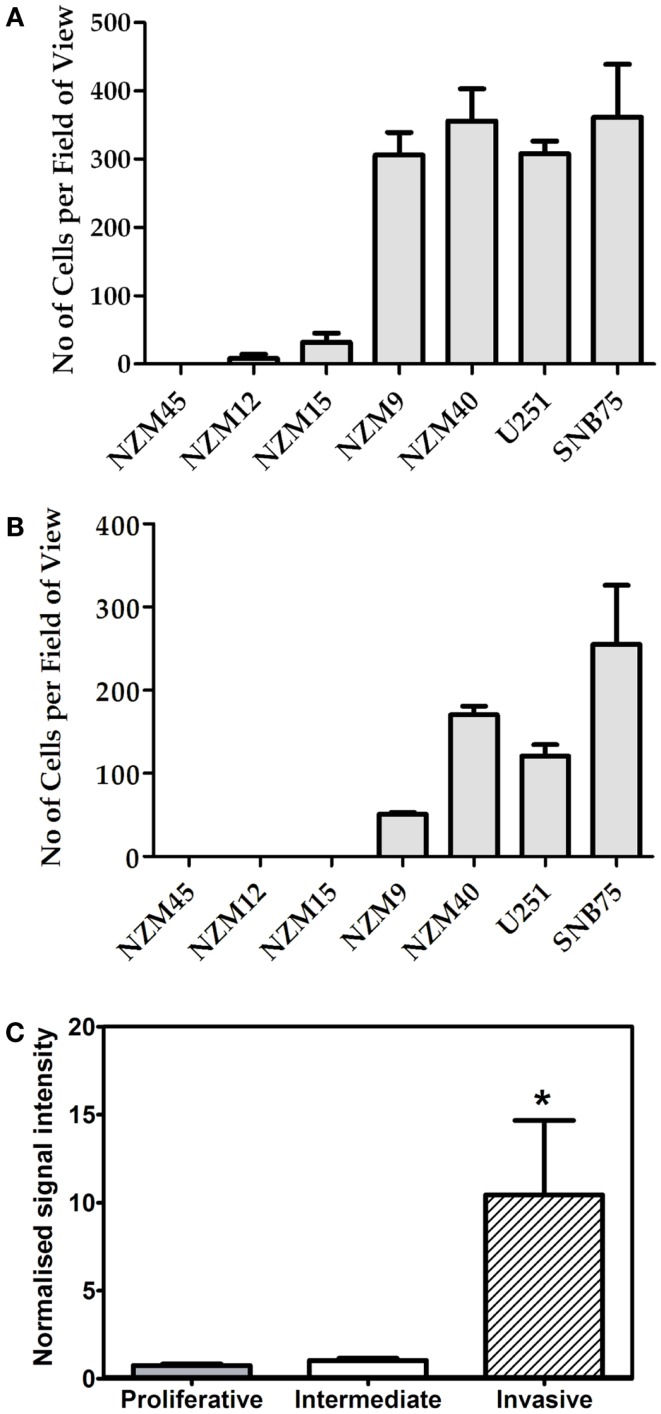
**GLIPR1 cell migration and invasion**. Frequency with which melanoma and glioma cells cross a pored membrane by **(A)** migration or **(B)** invasion was assessed by microscope after 24 h. Number of migrating or invading cells was calculated by counting the number of cells per field of view in 25 microscopic fields per well. Data shown as the average number of cells per field of view ± SEM from three **(A)** or two **(B)** independent experiments respectively. **(C)** GLIPR1 transcript levels were significantly higher in an independent panel of cell lines with experimentally validated invasive potential (20).

Having confirmed variable expression of GLIPR1, we then chose to investigate the relationship between GLIPR1 abundance and invasion in melanoma and glioma cells. *In vitro* cell migration and invasion was positively correlated with endogenous GLIPR1 expression levels (*r*^2^ = 0.94 and *r*^2^ = 0.91 respectively, Figures 2A,B; Figure S4 in Supplementary Material) suggesting a role for GLIPR1 in the migratory or invasive potential of melanoma cells. Cells with the highest GLIPR1 expression (NZM9, NZM40, U251, SNB75) showed the highest number of migrating and invading cells (Figures 2A,B). Conversely, cells with relatively low levels of GLIPR1 (NZM12, NZM15, NZM45) showed little to no migrating cells and no detectable invasion. The total number of cells invading through the Matrigel matrix was less than in the absence of matrix, but the overall trend of invasion was similar to that of migration: cell lines with highest GLIPR1 levels (NZM40 and SNB7) showed the highest invasion. Increased GLIPR1 transcript levels were also associated with increased invasion in an independent set of publically available microarray data generated from melanoma cell lines with experimentally validated invasive potential (Figure 2C) (20).

### GLIPR1 knockdown caused reduced cell invasion and proliferation

**Figure 3:**
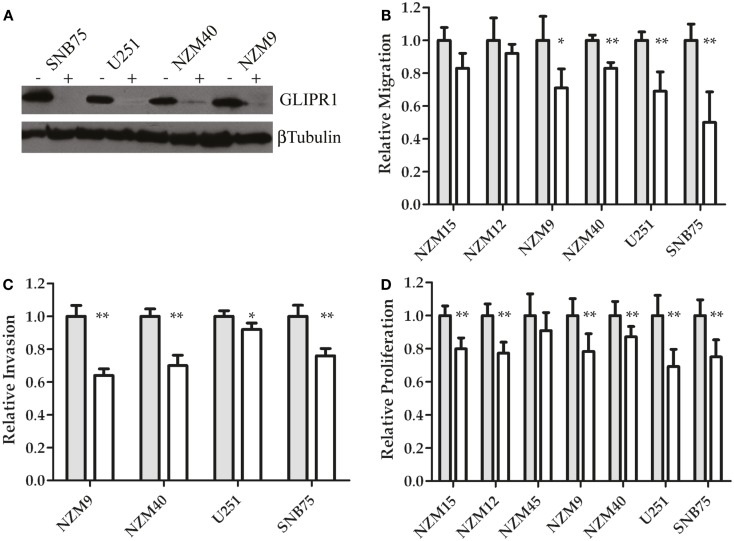
**GLIPR1 siRNA knockdown decreases cellular invasion and proliferation**. **(A)** GLIPR1 protein levels following knockdown were determined by western blotting (40 μg of total protein per lane) from cells 72 h following transfection with siRNA. GLIPR1 knockdown with siGLI (+) and control treatment with non-targeting siRNA siNT (−) are indicated above gel. Low levels of endogenous GLIPR1 prevented assessment of the extent of knockdown in NZM15, NZM12, and NZM45 by western blotting. Relative migration **(B)** and invasion **(C)** of cells across the membrane of transwell inserts was measured 24 h after siGLI. Data shown as the average number of cells per field of view ± SEM from three **(B)** or two **(C)** independent experiments. Error bars indicate SEM; *p < 0.005, **p < 0.001. No migration was observed for NZM45 (Figure 2A) and no invasion was seen for NZM45, NZM12, or NZM15 (Figure 2B). **(D)** Cell proliferation was quantified using MTT-based colorimetric assay. Results are mean of two independent experiments, *n* = 4. Results in **(D)** are shown as data for cells 4 days after transfection with siGLI relative to data for cells transfected with siNT. Results for **(B,C)** are shown for cells 24 h following transfection.

To further investigate the relationship between GLIPR1 expression and cell migration and invasion, GLIPR1 expression was decreased using siRNA. siRNA-mediated knockdown of GLIPR1 resulted in a significant decrease in the number of melanoma and glioma cells migrating across the membrane relative to non-targeting controls (Figure 3; Figure S5 in Supplementary Material). We used glioma cell lines in which GLIPR1 had previously been shown to modulate invasive behavior (11) as positive controls to compare with melanoma cell lines in our *in vitro* invasion assays. SNB75 glioma cells, with the highest pre-knockdown migration rate, showed about a 50% decrease in cell migration 24 h after GLIPR1 knockdown (Figure 3B). Similarly, the high GLIPR1-expressing cell lines NZM40, NZM9 and U251 showed a 20–30% decrease in migration compared to non-targeting controls. Cells with lower GLIPR1 levels (NZM12, NZM15) showed no measurable change in the already small number of cells migrating across the membrane after knockdown which reflects the intrinsically weak invasive potential of these cells.

GLIPR1 knockdown led to a significant reduction in the number of cells invading through Matrigel matrix (Figure 3C). The largest decrease in invasion was observed in NZM9 cells (∼38%) followed by NZM40 (30%) and SNB75 (24%) cells, with U251 cells (8%) showing a small but significant reduction in cell invasion (Figure 3C). Overall, the results of our Transwell assays and knockdown experiments support the notion that GLIPR1 is involved in mediating the invasive potential of melanoma cell lines.

siRNA-mediated knockdown of GLIPR1 resulted in a 10–22% decrease in proliferation in melanoma cells compared to 31 and 25% decrease for U251 and SNB75 glioma cells respectively, 4 days after transfection (Figure 3D). Reduced proliferation in glioma cell lines was observed by Rosenzweig et al. (11) who also reported that silencing of GLIPR1 induced apoptosis in some glioma cells. However, we saw no evidence of increased apoptosis or cell cycle-related growth arrest in any of the glioma and melanoma cells tested in this study (data not shown).

### Cellular localization of GLIPR1

To help understand how GLIPR1 could mediate invasion we examined cellular localization. GLIPR1 contains a predicted signal peptide and C-terminal TMD suggesting it may be translocated to the endoplasmic reticulum (ER) and trafficked to the cell surface as an integral membrane protein. *In vitro* translation assays showed GLIPR1 is processed to a higher molecular weight form in the presence of ER membranes (Figure S6A in Supplementary Material). This higher molecular weight form is sensitive to Endoglycosidase H digestion, indicating that it is glycosylated (Figure S6B in Supplementary Material). GLIPR1 segregates in the pellet fraction upon sodium carbonate extraction (Figure S6C in Supplementary Material), which confirms that it is integrated in the membrane. The higher molecular weight glycosylated GLIPR1 is protected from protease digestion in the absence of detergent (Figure S6D in Supplementary Material). Taken together these results indicate that GLIPR1 has a functional signal peptide and TMD, and is translocated into the ER where it is glycosylated at a site in its ER-lumen exposed soluble domain. Cell surface biotinylation assays in GLIPR1-expressing NZM9 cells demonstrated that GLIPR1 is present at the cell surface (Figure S7 in Supplementary Material).

### GLIPR1 expression in melanoma and skin tissue

**Figure 4:**
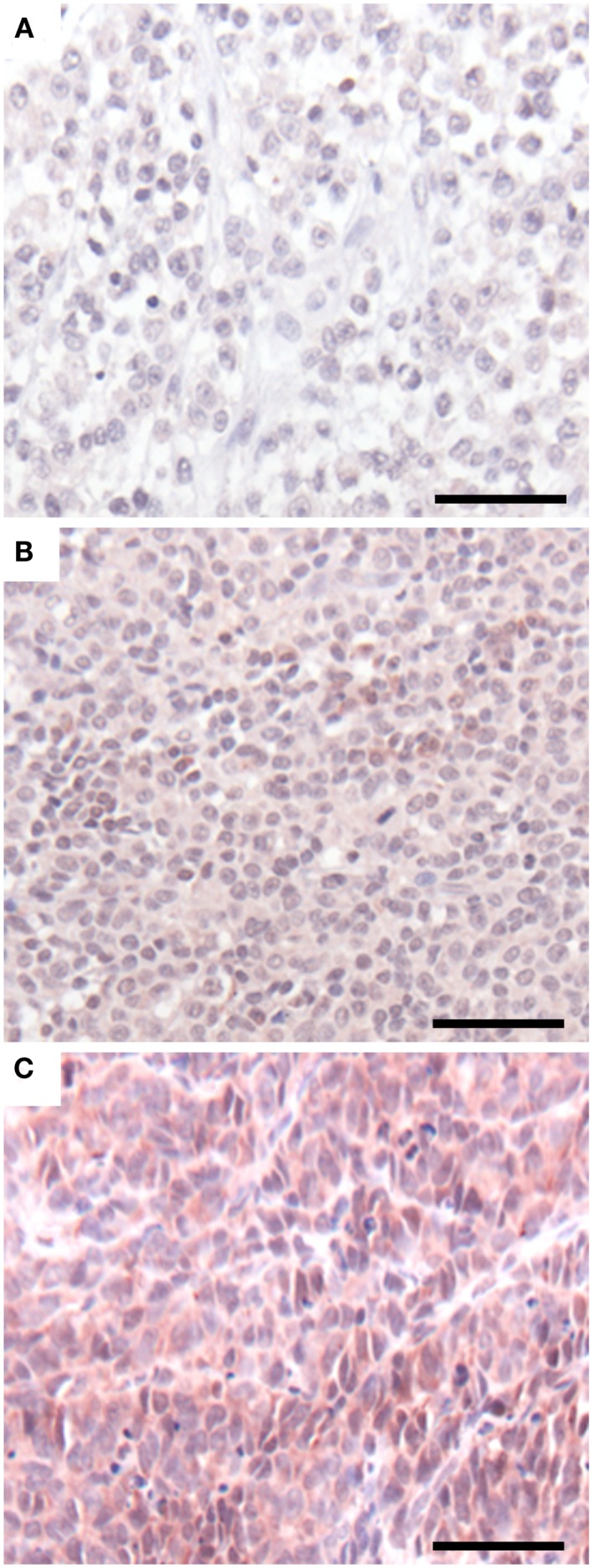
**GLIPR1 staining in malignant melanoma**. GLIPR1 immuno-staining in **(A)** malignant melanoma of the heel (+, low staining intensity), **(B)** malignant melanoma of the pate/crown (++, moderate staining intensity), and **(C)** malignant melanoma of the thumb (+++, high staining intensity). GLIPR1 immuno-positive regions were stained with NovaRED. All images were photographed at a power of 200×. Scale bar = 50 μm. Samples shown are from Melanoma tissue microarrays (US Biomax, Inc.).

**Figure 5:**
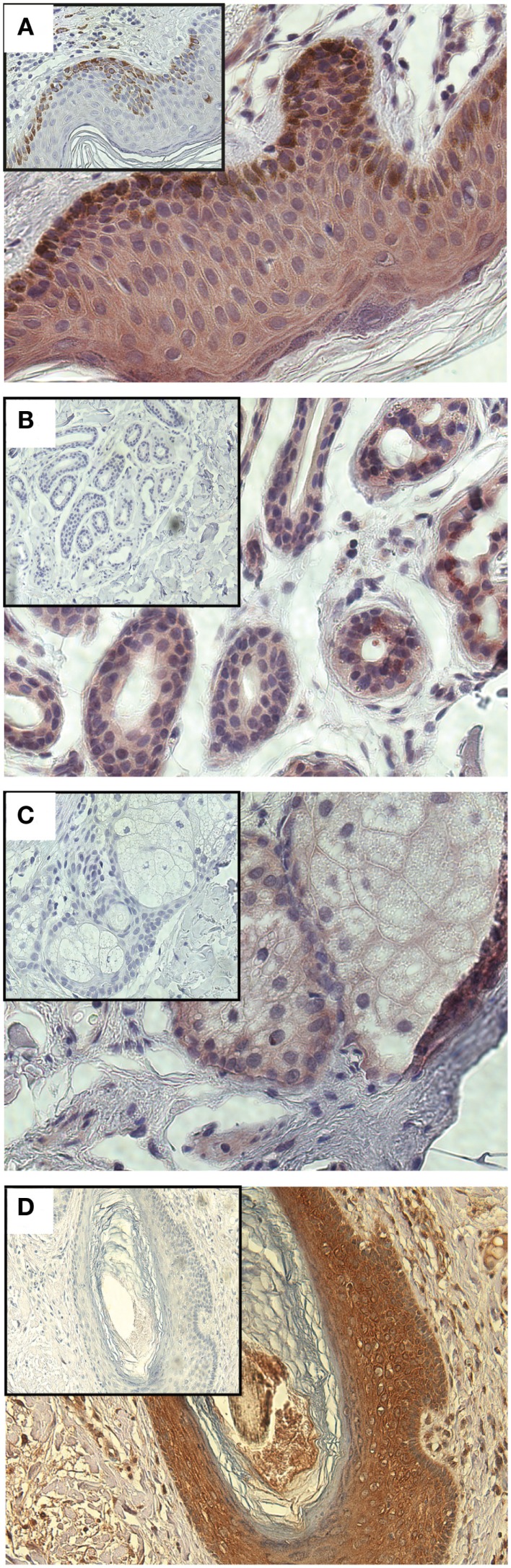
**Immunohistochemical staining of GLIPR1 in skin specimens**. Immuno-reactive cells of normal skin **(A)**, sweat glands **(B)**, sebaceous glands, **(C)**, hair follicles **(D)**. Skin specimens **(A–C)** were stained with NovaRED to give a red color for GLIPR1 immuno-positive regions, while hair follicles **(D)** were stained with DAB to give brown color for GLIPR1. Cell nuclei are stained blue with hematoxylin. Inset images show negative control reacted with the non-specific goat serum instead of anti-GLIPR1 antibody. Lack of brown staining in the negative control indicates this is due to GLIPR1 and not melanin. All images were photographed at a power of 400×.

Having demonstrated a relationship between GLIPR1 expression and migration/invasion in melanoma cell lines, we investigated GLIPR1 expression in melanoma and skin tissue samples. Immunohistochemical staining of malignant melanoma tissue samples showed variable expression of GLIPR1 (Figures 4A–C) in a similar fashion to the NZM cell lines. Of the 76 melanoma specimens analyzed, 50% showed moderate to high immuno-reactivity (++ and +++) for GLIPR1. The other 50% of the specimens (38/76) showed either no (26/76) or low (12/76) staining for GLIPR1. However, unlike glioma, there was no obvious relationship between GLIPR1 positivity and melanoma progression. In normal skin, all layers of the epidermis except for the stratum corneum were immuno-positive for GLIPR1 (Figure 5A). Cells of the basal layer showed positive staining for GLIPR1 (Figure 5A, red). Most of the fibro-elastic tissue in the dermal layer of skin was found to be immuno-negative for GLIPR1. However, merocrine sweat glands, sebaceous glands, and hair follicles within the dermis were found to be immuno-positive for GLIPR1 (Figures 5B–D).

## Discussion

*GLIPR1* transcript and protein have been reported in various tissues including heart, lung, liver, spleen, skin, colon, pancreas, lymphocytes, muscle, bone marrow, placenta, adrenal gland, prostate, glioma, and prostate cancer (5, 11, 21). More recently, *GLIPR1* has been found to be differentially expressed in ovarian cancer and acute myeloid leukemia (7, 22). However, GLIPR1 expression analysis in melanoma has not been previously reported. Using RT-qPCR and western blots we found variable expression of *GLIPR1* mRNA and protein in different metastatic melanoma cell lines with two melanoma cell lines having similar levels to glioma cell lines previously reported as having high GLIPR1 expression (Figures 1A,B). These data add to the growing number of cancer cell types in which GLIPR1 has been reported to be variably expressed.

Epigenetic modulation of GLIPR1 expression by promoter methylation has been reported previously in prostate cancer, glioma, and Wilms’ tumors, and we show that it is also the case in melanoma cells in the samples tested, with decreasing promoter methylation associated with increasing levels of GLIPR1. Our study focused on metastatic melanoma cells. Whether GLIPR1 promoter methylation is dynamic during disease progression or shows higher or lower levels of methylation in metastatic melanoma cells compared to normal melanocytes, benign nevi and primary melanoma remains unknown and requires further investigation.

We investigated the relationship between endogenous GLIPR1 levels and migration and invasion potential *in vitro*, which revealed that melanoma cells with higher endogenous GLIPR1 levels displayed significantly greater migration and invasion capability than cells with relatively lower levels of GLIPR1. siRNA-mediated knockdown of GLIPR1 inhibited migration and invasion in melanoma cell lines (Figures 3B,C). The low level of migration and complete lack of invasion in melanoma cells with relatively lower GLIPR1 levels (NZM15, NZM12, and NZM45) suggests that a threshold level of GLIPR1 expression may be present in melanoma cells with a strongly invasive phenotype as GLIPR1 levels in NZM9, NZM40, U251, and SNB75 cells after siRNA-mediated knockdown were still higher than that in untreated and weakly invasive NZM15, NZM12, and NZM45 cells. Overexpression of GLIPR1 has previously been shown to increase invasion of several glioma cell lines (11), and is consistent with our findings in melanoma cells suggesting GLIPR1 acts as an oncoprotein rather than a tumor suppressor in melanoma. GLIPR1 knockdown caused a modest but reproducible decrease in proliferation in both melanoma and glioma cells, suggesting that GLIPR1 may play a role in cell growth at some level. It is possible that the reduced invasion we observed after GLIPR1 knockdown was a consequence of the observed reduction in proliferation.

We demonstrated for the first time that GLIPR1 contains a functional signal peptide and TMD (Figure S6 in Supplementary Material). We confirm that it is a glycoprotein, most likely at the predicted glycosylation site at asparagine 92 (5), which is shown by homology modeling of GLIPR1 to be on the surface of the protein (data not shown). Glycosylation was also seen in the recent expression and structural studies by Asojo and co-workers (23, 24). GLIPR1 is translocated to the cell surface where its soluble N-terminal domain will be exposed to the extracellular space. The exposure of GLIPR1’s soluble domain to the extracellular matrix is consistent with a role of GLIPR1 in invasion and migration. Future investigations as to whether GLIPR1 possesses proteolytic activity as displayed by another member of the CAP family (25) would prove invaluable and provide insight as to whether such an activity is directly associated with the cell invasion and migration properties similar to that of matrix metalloproteases.

The present study characterized GLIPR1 expression by immunohistochemistry in normal and cancerous skin. Previously, mRNA levels of *GLIPR1* transcripts in skin have been reported as very low or undetectable (11, 21). Immunohistochemistry showed GLIPR1 was detectable in normal skin (Figure 5) within certain tissues such as epithelial cells of epidermis, sebaceous glands, merocrine sweat glands, and hair follicles. Immunohistochemical staining of melanoma tissues confirmed variable expression of GLIPR1, similar to that observed in NZM cell lines, with almost an equal proportion of melanoma specimens expressing relatively higher or relatively lower levels of GLIPR1. It does not appear that GLIPR1 levels are uniformly elevated with increasing melanoma stage, which is again consistent with GLIPR1 being sensitive to dynamic gene expression changes that are associated with the postulated phenotype switching capacity of melanoma cells during disease progression. Although we demonstrated variable GLIPR1 staining in melanoma tissue microarray samples, these samples lacked clinical information, so the clinical significance of variable GLIPR1 expression remains unknown. A survey of publically available array data shows that GLIPR1 levels are variable in melanoma (13), which is in agreement with our observations in this study. However, there is little useful published melanoma array data that includes patient information to determine if GLIPR1 transcript abundance is correlated with clinical outcome. We analyzed GLIPR1 levels in the study of metastatic melanoma samples by Bogunovic and colleagues (26), which includes clinical data, and found that elevated GLIPR1 levels were significantly positively correlated with survival (Figure S8 in Supplementary Material). Given elevated GLIPR1 levels are part of a multi-gene expression signature that is associated with a phenotypic balance between invasive and proliferative states in melanoma, we speculate that metastatic melanomas with an invasive phenotype may proliferate more slowly (according to the phenotype switching model), and thus offer a survival advantage to the patient, compared to less invasive but more rapidly proliferating tumors. Further data are required to confirm this observation. Yang and co-workers (27) identified *GLIPR1* as part of a group of extracellular matrix-related genes involved in a melanocyte growth arrest program, which further implicates GLIPR1 as having a potential role in the dynamics of melanocyte biology via interaction with the extracellular microenvironment. Further investigation is required to clarify this.

The MITF and POU3F2 (BRN2) transcription factors have been reported to be inversely correlated in melanoma cells (28), are both key markers of the phenotype switching model of invasive potential [reviewed in (29)], and are both possible regulators of GLIPR1. GLIPR1 is inversely correlated with MITF in NZM cell lines (13), suggesting that perhaps MITF might negatively regulate GLIPR1. However, the evidence to support this is lacking: there are no MITF binding sites in the GLIPR1 promoter (not shown), and recent studies by others – although not taking epigenetic silencing into account – did not identify GLIPR1 as a direct target of either MITF or POU3F2 in melanoma cells (30–32). Based on our GLIPR1 promoter methylation data, we speculate that perhaps genome-wide epigenetic re-programing can occur in melanoma cells, to which GLIPR1 is sensitive, and that is associated with phenotype switching mechanisms in melanoma cells.

Malignant melanoma is an aggressive and unpredictable cancer. Currently there is no cure for metastatic melanoma, and no way of determining which patients will respond to current treatment options. The mechanisms underlying melanoma progression and resistance to therapeutic agents are not well understood. There are few treatment options once metastasized, and new biomarkers that aid diagnosis, predict clinical outcome, and suggest new therapies are required. Based on the data presented here, future studies will focus on identifying whether GLIPR1 levels and/or promoter methylation status may be a clinically beneficial marker of metastatic melanoma phenotype.

## Conflict of Interest Statement

The authors declare that the research was conducted in the absence of any commercial or financial relationships that could be construed as a potential conflict of interest.

## Supplementary Material

The Supplementary Material for this article can be found online at http://www.frontiersin.org/Cancer_Genetics/10.3389/fonc.2013.00225/abstract
