# Supplementary material for: Variable Expression of GLIPR1 Correlates with Invasive Potential in Melanoma Cells
Source: Front Oncol. 2013 Aug 30;3:225. doi: 10.3389/fonc.2013.00225 (PMC3757444; doi:10.3389/fonc.2013.00225)
Supplement: Supplementary file 1 [file 47595_Tyndall_DataSheet1.PDF]

**Glioma immunohistochemistry**

To validate the performance of the antibody in skin and melanoma samples, we performed immunohistochemistry on brain tissues (Fig. S6) previously reported to show relatively higher GLIPR1 expression with advancing tumour grade [1].

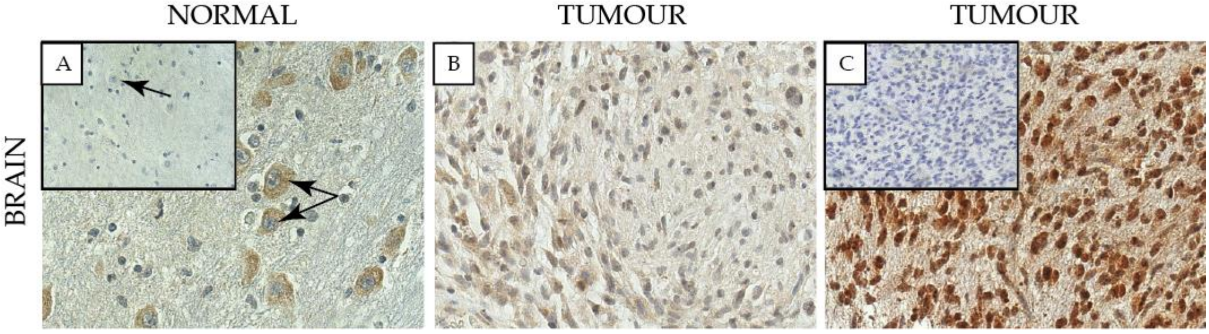


**Figure S1** Panels (A-C) indicate IHC of normal and cancerous brain tissue. GLIPR1 immuno-positive regions were stained with DAB (brown colour) in glioma specimens. Cell nuclei are stained blue with haematoxylin. Neurons are marked with arrows in panel (A). Insets show negative controls incubated with the non-specific goat serum instead of anti-GLIPR1 antibody. All images were photographed at a power of ×400.

**Results**

Immunohistochemical staining of normal brain showed neuron-specific immuno-reactivity (Figure S1A) compared to stronger and more widespread staining observed for GLIPR1 in gliomas (Figure S1B-C), in accordance with previously reported up-regulation in gliomas [1-3]. In our glioma samples GLIPR1 staining increased with advancing tumour stage (Figure S1B-C).

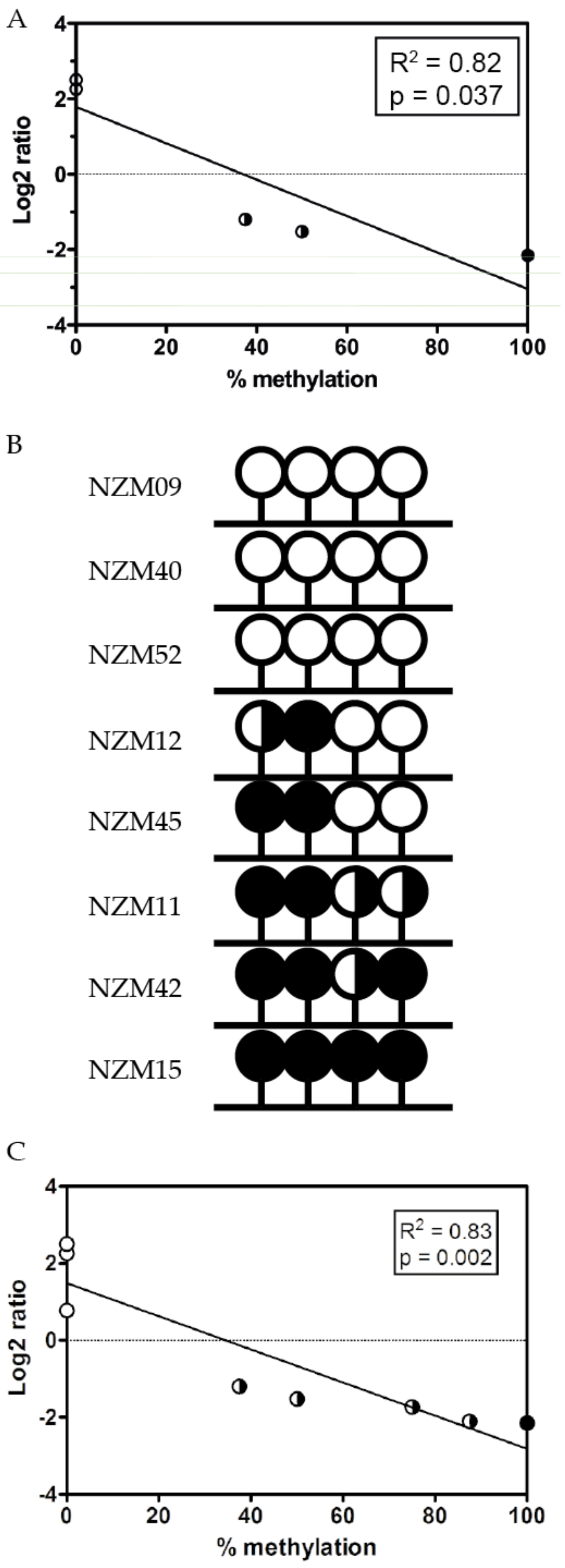
A
$R^2$ = 0.82
p = 0.037
Log2 ratio
% methylation
B
NZM09
NZM40
NZM52
NZM12
NZM45
NZM11
NZM42
NZM15
C
$R^2$ = 0.83
p = 0.002
Log2 ratio
% methylation

**Figure S2** (A) Relative GLIPR1 transcript levels, measured by using microarrays [4], were negatively correlated to promoter methylation status in melanoma cells ($r^2$ = 0.82, p = 0.037). Cell lines and key as in Fig. 1A. (B) Summary of bisulfite sequencing data showing GLIPR1 CpG promoter methylation status for melanoma cell lines with known invasive potential (this study and [4]). NZM09, NZM40 and NZM52 are strongly invasive compared to the remaining cell lines. Lollipops represent individual CpG dinucleotides within a CpG island in the GLIPR1 promoter. DNA from three different vials of each NZM cell line was sequenced at least twice on both strands. White, unmethylated; black = methylated; black/white = hemimethylated. (C) Relative GLIPR1 transcript levels, measured by using microarrays [4], were negatively correlated to promoter methylation status in melanoma cells ($r^2$ = 0.83, p = 0.002). Cell lines and key as in (B).

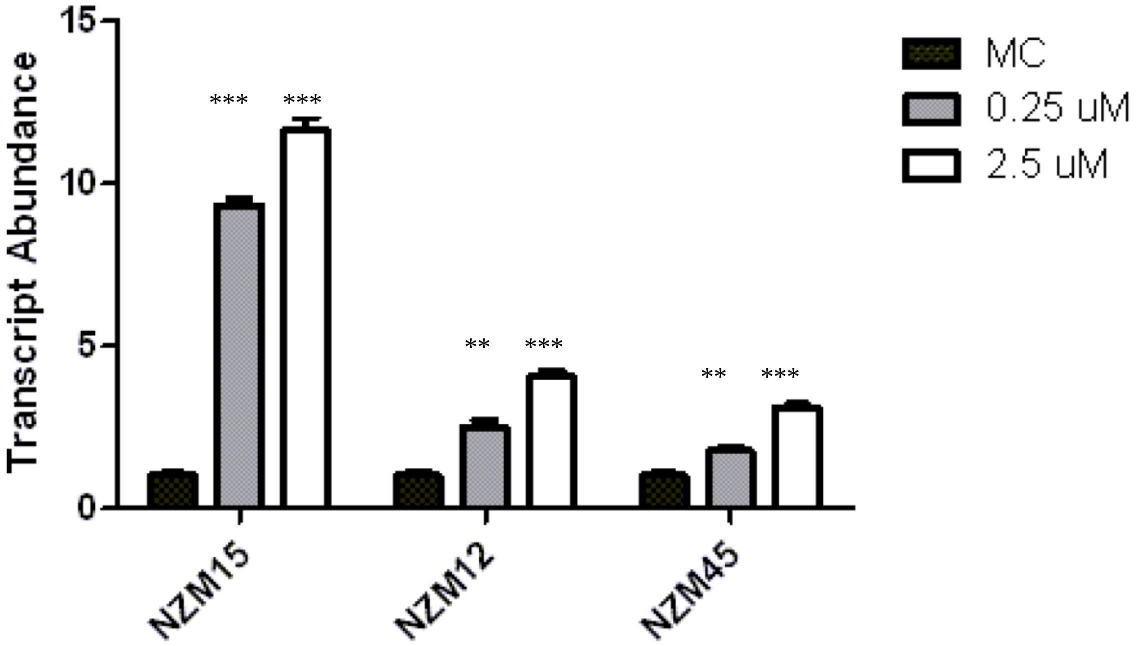


**Figure S3: Azacytidine induced increased expression of GLIPR1**. NZM cells with relatively low GLIPR1 levels and some degree of promoter methylation were treated with 0.25 µM or 2.5 µM 5-azacytidine for 48 hours (n = 3). RNA isolation and RT-qPCR was performed as described in the Methods. Data was analysed using qbasePLUS (Biogazelle). Results are shown as normalised transcript abundance relative to the media-only control (MC = 1.0) for each cell line. 5-azacytdine was redissolved in cell culture media. Error bars are SEM, ** $p < 0.01$, *** $p < 0.001$ (Unpaired *t* test).

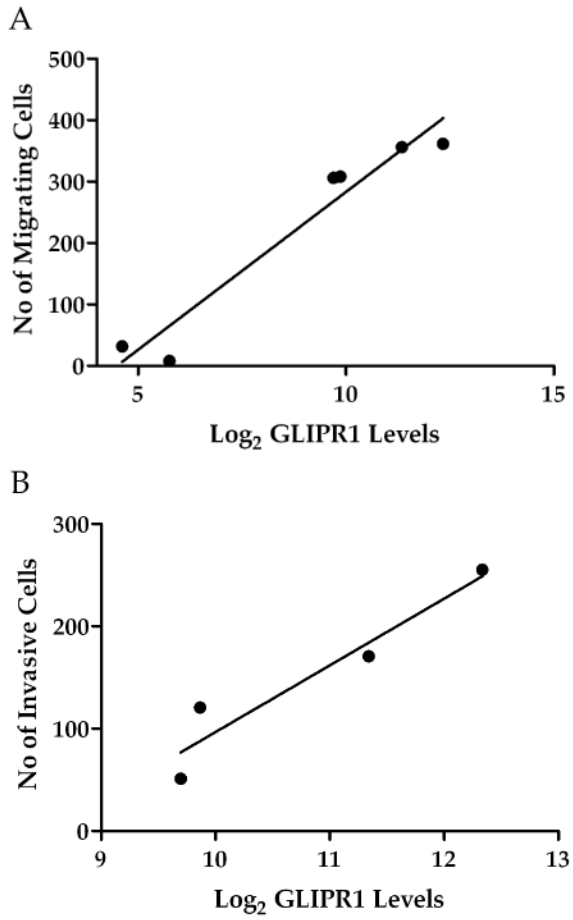


**Figure S4**: Linear regression between $\log_2$ endogenous GLIPR1 expression levels and number of (A) migratory ($r^2 = 0.939$) and (B) invasive ($r^2 = 0.911$) cells respectively. The linear regression plot for migration (S2*A*) does not include NZM45 as it did not show any migration and the plot for invasion (S2*B*) does not include NZM15, NZM12 and NZM45 cells since they showed no invasive activity.

**siRNA validation**

To investigate the effect of GLIPR1 on cancer cell proliferation, migration and invasion, we used siRNAs targeted against GLIPR1 mRNA. Transfection of cells with siRNA for 24 h resulted in a substantial decrease in the *GLIPR1* transcripts measured by RT-qPCR (Fig. S3). Cells with higher levels of endogenous *GLIPR1* showed a larger decrease in final *GLIPR1* levels than did the cells with lower levels. The lowest amount of knockdown was observed for NZM15 and NZM45 cells (~46%) which remained consistent even when transfected with higher concentration of siRNA (up to 80 nM).

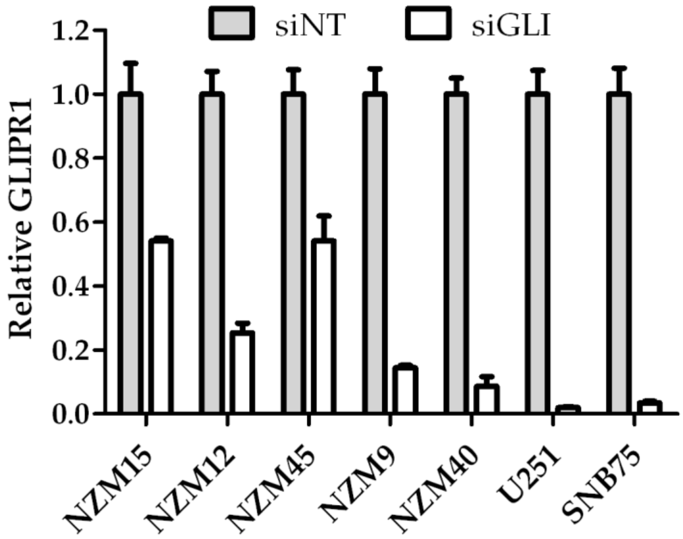


**Figure S5: siRNA mediated GLIPR1 knockdown** in glioma and melanoma cells 24 h after transfection. siGLI refers to cells transfected with 10 nM of siRNA targeted against GLIPR1 and siNT refers to cells transfected with non-targeting siRNA. Results shown as transcript levels of siGLI relative to siNT. Results are mean of three independent experiments; error bars indicate SEM.

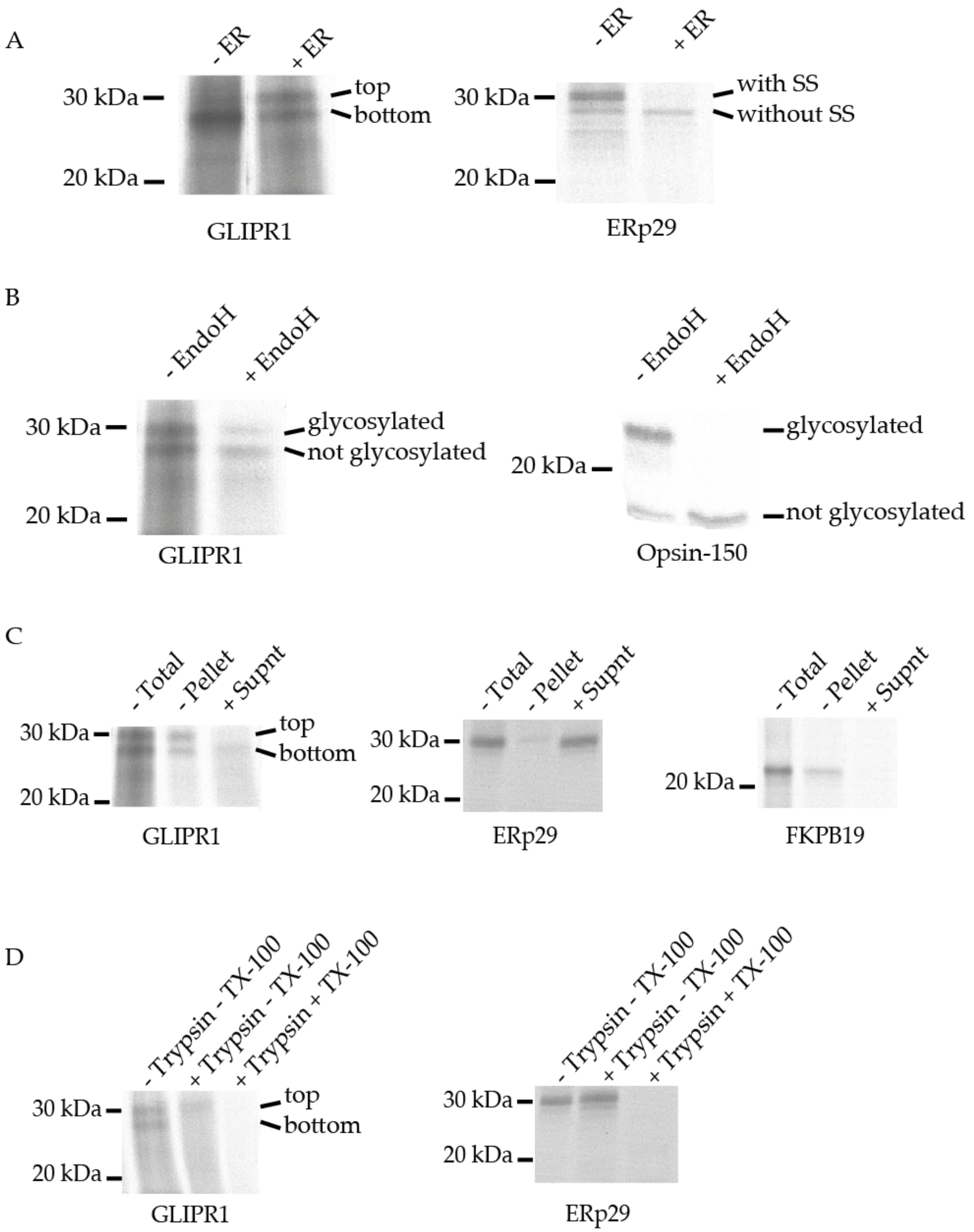


**Figure S6 Translation: GLIPR1 is an N-glyosylated integral membrane protein.** GLIPR1 and control mRNAs were translated *in vitro* in the presence of $^{35}$S- methionine and tested by a series of assays for ER-associated characteristics. The radio-labeled proteins obtained were separated on SDS PAGE gels and detected by exposure to imaging film. (A) GLIPR1 and the

ER luminal protein ERp29 were translated with or without an ER membrane preparation. Both proteins show evidence of processing when ER membranes are included in the reaction. Unlike ERp29 which shows a decrease in molecular weight consistent with the cleavage of its signal sequence upon import into the ER, GLIPR1 shows an increase in molecular weight upon import into the ER. (B) GLIPR1 and the N-terminal 150 amino acids of opsin were translated in the presence of an ER membrane preparation, then incubated with and without EndoH, an enzyme which specifically cleaves ER- glycosylated Asparagine residues. Opsin, a well-documented N-glycosylated protein, was used as a positive control in this experiment. The ER resident forms of both GLIPR1 and opsin show sensitivity to EndoH, indicating that GLIPR1, like opsin, is modified by glycosylation in the ER. (C) GLIPR1, ERp29 (a soluble protein) and FKBP19 (an integral membrane protein) were translated in the presence of an ER membrane preparation, then extracted with sodium carbonate (pH 11.3). Membrane-associated material was isolated by centrifugation (Pellet), the supernatant TCA-precipitated (Supnt), and these were both compared with an untreated sample (Total). ERp29 partitions in the supernatant fraction, consistent with its soluble nature. GLIPR1 partitions in the pellet fraction, in the same fashion as FKBP19, indicating that GLIPR1 is an integral membrane protein. (D) GLIPR1 and ERp29 were translated in the presence of an ER membrane preparation, then digested with Trypsin TPCK in the presence or absence of TX-100. ERp29 is protected from protease digestion in the absence of detergent, consistent with its localisation in the ER lumen. GLIPR1's top band (N-glycosylated transmembrane form) is protected from protease digestion in the absence of detergent, whereas the bottom band is not. Together with the EndoH and the sodium carbonate extraction data, this indicates that GLIPR1's top band corresponds to the membrane integrated form, with its soluble domain exposed to the ER lumen, whereas the bottom GLIPR1 band is likely to correspond to improperly ER targeted protein most likely aggregated in the cytosol.

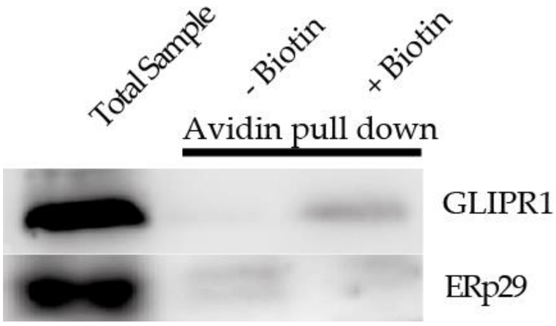


**Figure S7: GLIPR1 is expressed on the cell surface**. NZM9 cells were incubated with (+ Biotin) and without (- Biotin) membrane-impermeant EZ-Link Sulfo-NHS-LC biotin and lysed, then the biotinylated proteins were pulled down with UltraLink Immobilised Neutravidin. Total sample and pulled down proteins were analysed by immunoblotting with anti-GLIPR1 and anti-ERp29 (used as a negative control, due to its lack of exposure to the cell surface). This shows that a proportion of GLIPR1 is localised on the cell surface.

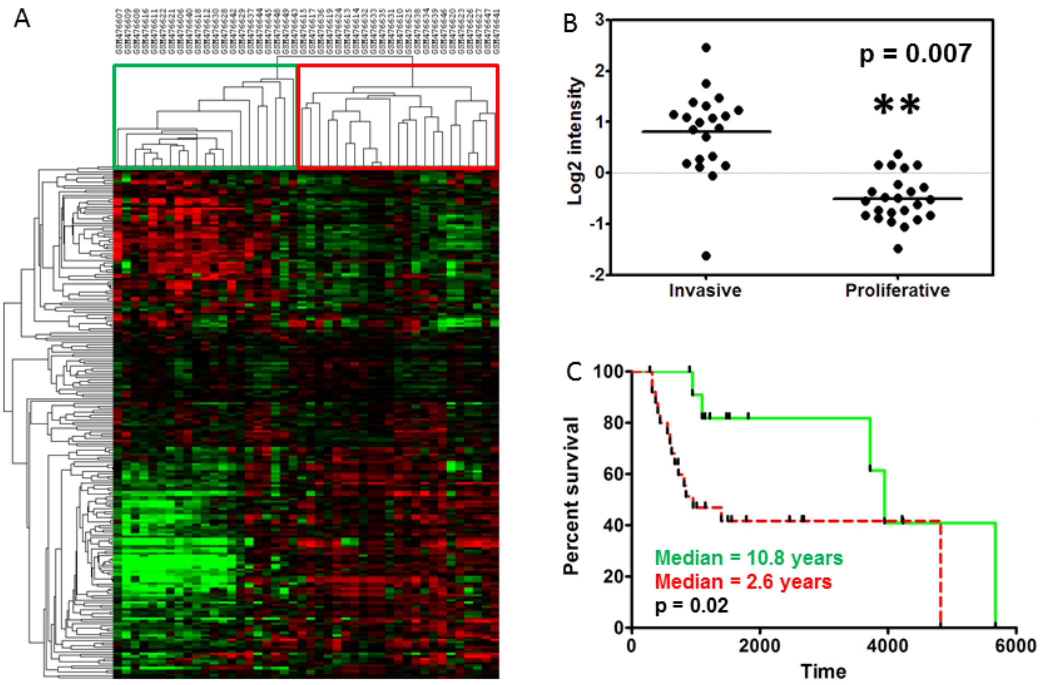


**Figure S8:** Semi-supervised hierarchical clustering of metastatic melanoma samples. (A) Raw data from the Bogunovic et al. [5]; GEO accession GSE19234) was normalised and filtered by using BRB ArrayTools (developed by Dr. Richard Simon and BRB-ArrayTools Development Team) to include only probe set identifiers from the 96-gene invasive signature reported in [5]. The normalised, filtered, log2-transformed gene list was submitted to GenePattern [6] for hierarchical clustering by using Pearson correlation with average linkage on median-centred data. The resulting two major clusters corresponded to a more invasive gene expression signature with elevated GLIPR levels (left cluster, green box), or a less invasive, more proliferative signature with lower GLIPR1 levels (right cluster, red box). (B) GLIPR1 levels were significantly higher in samples from the invasive compared to the proliferative cluster [green and red, respectively, in (A)]. (C) Median survival was significantly shorter in patients from the proliferative signature cluster with relatively lower GLIPR1 levels (red) compared to the invasive signature cluster with higher GLIPR1 levels (green).

## Supplementary Materials and Methods

### *In vitro* transcription and translation

PCR products spanning the promoter and the ORF region of GLIPR1, ERp29, FKBP19 and opsin (150 N-terminal amino acids) were amplified from plasmids [GLIPR1, GenBank BC012510 (IMAGE: 4453841); ERp29, GenBank BM455050 (IMAGE 5499959); FKBP19, GenBank BC037596 (IMAGE 5250344); opsin, pGEM3z-opsin-cys-null, a kind gift from Prof S. High, Faculty of Life Sciences, The University of Manchester, Manchester, UK], using the following primers GLIPR1 forward ACC ATT AGG CCT ATT TAG and reverse GTC CAA AAG AAC TAA ATT AG, ERp29 forward ACC ATT AGG CCT ATT TAG and reverse CAG CTC CTC TTT CTC GGC; FKBP19 forward ACCATTAGGCCTATTTAG and reverse TTTCTTTTTGCTCTTGTTTGGTTTCTC; opsin forward ATTACGAATTTAATACGACTCACTATAGGG and reverse CTCCCCGAAGCGGAAGTTG, respectively. The PCR products were used as templates for *in vitro* transcription, using T7 RNA polymerase (Promega) or SP6 RNA polymerase (New England Biolabs), as described by the manufacturer. The RNA obtained was purified with an RNeasy Mini Kit (Qiagen). The RNAs were translated in rabbit reticulocyte lysate (Promega) with $^{35}$S-methionine (EasyTag Express $^{35}$S Protein Labelling Mix; Perkin Elmer), in the presence or absence of ER semi-permeabilised cells. Semi-permeabilised Hela cells were prepared as previously described [7]. Briefly, Hela cells were permeabilised with digitonin (Calbiochem) at 20 µg/ml and endogenous RNA was removed with micrococcal nuclease (Sigma) treatment. Translations were carried out for 20 min at 30$^{o}$C. Translation initiation was then inhibited with 2.5 mM aurintricarboxylic acid (ATA; Sigma), and chain elongation continued for a further 5 min at 30$^{o}$C. Translation was then completely inhibited by treatment with 1 mM puromycin (Sigma) and 5 mM EDTA. In the case of the translations performed in the presence of semi-permeabilised cells, the membrane fraction was isolated by centrifugation at 10,000g for 10 sec, washed with KHM (110 mM KOAc, 2 mM MgOAc, 20 mM HEPES.KOH pH 7.2) and re-suspended in KHM. The occurrence of N-glysosylation was tested by Endoglycosidase H (EndoH; New England Biolabs) digestion after denaturation of the samples, using conditions described by the manufacturer. The integration of proteins in the membrane was tested by sodium carbonate extraction of the membrane fraction. The membrane pellet obtained at the end of the translation in presence of ER membranes was re-suspended in a freshly prepared solution of 0.1 M $Na_2CO_3$ pre-cooled on ice, incubated on ice for 15 min,

and centrifuged at 117000 g at 4ºC in a TLA 100 rotor. This pellet was re-suspended in SDS PAGE sample buffer, whereas the supernatant was precipitated with 1/10 volume of 100% TCA and the ensuing pellet also re-suspended in SDS PAGE sample buffer. The orientation of membrane proteins across the membrane was tested by a protease protection assay. Trypsin protection was used to identify the ER *in vitro* translation products accessible to the cytosol, and therefore not protected by the ER membrane. The membrane pellet obtained following translation in the presence of ER membranes was re-suspended in KHM and supplemented with either 3 µl of water, 3 µl of TPCK Trypsin (Sigma) at 1 mg/ml and 1.7 µl of 500 mM EDTA, or 3 µl of TPCK Trypsin at 1 mg/ml, 1.7 µl of 500 mM EDTA and 3 µl of TX-100 at 10% v/v. After 30 min incubation on ice, conc. 1 mM PMSF (Sigma) and SDS PAGE sample buffer were added. All samples derived from the *in vitro* translation reactions were analysed by SDS PAGE, the gels dried and exposed to imaging film (Kodak BioMax MR)

**Cell surface biotinylation assay**

NZM9 cells grown in 35 mm dishes were cooled on ice, and rinsed 3 times with ice-cold PBS, pH 8. Proteins were labelled with 1 ml of PBS pH 8 containing 1 mM EZ-Link Sulfo-NHS-LC biotin (Pierce) per well and incubated on ice for 30 min. The cells were then washed 4 times with 1 ml of ice-cold PBS, then lysed and collected in 1 ml of PBS containing 1% SDS, complete protease inhibitor (Roche) and 1U Benzonase (Sigma). The cell lysates (+/-biotin treatment) were incubated with 30 µl of UltraLink Immobilised Neutravidin (Pierce), and incubated for 2 h on a rock'n roller at room temperature. The samples were then centrifuged for 10 min at 10,000 g, and SDS PAGE sample buffer was added to the neuravidin bead pellet. GLIPR1 protein levels in the different samples were determined by western blotting and compared to a sample of total protein from a 35 mm dish. Endoplasmic reticulum protein 29 (ERp29), an intracellular protein, was used as a negative control.

**ERp29 Immunoblotting**

Serum from rabbits immunised with recombinant human ERp29 was used for detection of ERp29. Protein extracts were separated on SDS-PAGE and transferred onto nitrocellulose by a semi-dry transfer process. Membranes were blocked with 10% Sea Block (Pierce) in TBST, sequentially incubated with 1/2,000 anti-ERp29 and 1/20,000 goat anti-rabbit IgG (H+L)–HRP conjugate (Biorad), developed using SuperSignal West Pico Chemiluminescent Substrate (Thermo scientific) and imaged in a Fujifilm LAS-3000 Intelligent Dark Box.

***In vivo***

## Tissue specimens

Five µm sections of formalin-fixed, paraffin embedded tissue (skin, glioma and brain) on SuperFrost Plus slides were used for this study. In all 24 glioma sections were analysed (four grade-2 glioma, four grade-3 glioma and 16 grade-4 glioma sections). Of the 24 glioma specimens analysed in this study, 23 were supratentorial tumours (in the upper part of the brain, the cerebrum) and one was infratentorial (in the lowest part of the brain, the cerebellum)**.** The normal brain tissue was from the paraventricular cerebrum, normal brain near the corpus callosum containing chorid plexus, ependyma, grey and white matter.

### *Tissue arrays*

Prostate tissue microarrays were purchased from US Biomax, Inc. Prostate cancer tissue array with Gleason score (GS), grade and TNM staging information (catalogue number PR807) consisted of three cases of normal tissue, seven adjacent normal tissue (normal tissue in the vicinity of prostate cancer tissue), 44 cases of adenocarcinoma (four specimens with GS 2-4, 20 specimens each with GS 5-7 and 8-10) and 18 cases of hyperplasia.

## Immunohistochemistry

For prostate tissue specimens the percentage of GLIPR1 immuno-reactive nuclei was estimated by visual examination, as was staining of low, medium, or high intensity. For nuclear staining, a score of zero indicated no immuno-positive nuclei; a score 1 indicated <25% immuno-positive nuclei and a score 2 indicated >25% immuno-positive nuclei. Visualization of immuno-reactivity was achieved with diaminobenzidine (DAB) (Vector Laboratories) for glioma and brain tissue specimens. For glioma specimens, the overall immuno-staining score was designated as: 3 for >50% immuno-positive cells; 2 for >25 and <50% immuno-positive cells and 1 for <25% immuno-positive cells. For nuclear staining, a score of zero indicated no immuno-positive nuclei; 1 indicated <25% immuno-positive nuclei and 2 indicated >25% immuno-positive nuclei.

## References

1. Rosenzweig T, Ziv-Av A, Xiang C, Lu W, Cazacu S, et al. (2006) Related to testes-specific, vespid, and pathogenesis protein-1 (RTVP-1) is overexpressed in gliomas and regulates the growth, survival, and invasion of glioma cells. Cancer Res 66: 4139-4148.
2. Murphy EV, Zhang Y, Zhu W, Biggs J (1995) The human glioma pathogenesis-related protein is structurally related to plant pathogenesis-related proteins and its gene is expressed specifically in brain tumors. Gene 159: 131-135.
3. Rich T, Chen P, Furman F, Huynh N, Israel MA (1996) RTVP-1, a novel human gene with sequence similarity to genes of diverse species, is expressed in tumor cell lines of glial but not neuronal origin. Gene 180: 125-130.
4. Jeffs AR, Glover AC, Slobbe LJ, Wang L, He S, et al. (2009) A gene expression signature of invasive potential in metastatic melanoma cells. PLoS ONE 4: e8461.
5. Bogunovic D, O'Neill DW, Belitskaya-Levy I, Vacic V, Yu YL, et al. (2009) Immune profile and mitotic index of metastatic melanoma lesions enhance clinical staging in predicting patient survival. Proc Natl Acad Sci U S A 106: 20429-20434.
6. Reich M, Liefeld T, Gould J, Lerner J, Tamayo P, et al. (2006) GenePattern 2.0. Nat Genet 38: 500-501.
7. Wilson R, Allen AJ, Oliver J, Brookman JL, High S, et al. (1995) The translocation, folding, assembly and redox-dependent degradation of secretory and membrane proteins in semi-permeabilized mammalian cells. Biochem J 307 ( Pt 3): 679-687.
